# Plasmid-borne transcriptional regulator RamAp modulates *Salmonella* genes for environmental and host adaptation

**DOI:** 10.3389/fmicb.2026.1842592

**Published:** 2026-05-28

**Authors:** Yu-Ping Hong, Wen-Sheng Yeh, Mei-Hsiu Wan, Yung-Chieh Wu, Yen-Tong Chen, Wei-Kang Huang, Cheng-Hao Yang, Liang-Yu Su, Yi-Min Hong, Pei-Ju Tsai, Yu-Ting Chen, Ching-Ting Lin, Yi-Chyi Lai, Chien-Shun Chiou, Ying-Tsong Chen

**Affiliations:** 1 Ph.D. Program in Medical Biotechnology, National Chung Hsing University, Taichung City, Taiwan; 2 Centers for Disease Control, Taichung City, Taiwan; 3 Graduate Institute of Genomics and Bioinformatics, National Chung Hsing University, Taichung City, Taiwan; 4 School of Chinese Medicine, Graduate Institute of Chinese Medicine, China Medical University, Taichung City, Taiwan; 5 Biotechnology Center, National Chung Hsing University, Taichung City, Taiwan; 6 Department of Microbiology and Immunology, Chung Shan Medical University, Taichung City, Taiwan; 7 Institute of Molecular and Genomic Medicine, National Health Research Institutes, Miaoli County, Taiwan

**Keywords:** efflux pumps, flagellar, *Galleria mellonella* infection model, IS*Ecp1*, oxidative stress response, RamAp, transcriptional regulation, virulence factor

## Abstract

RamAp is a plasmid-borne homolog of the AraC family regulator RamA, commonly found on multidrug resistance (MDR) plasmids in *Salmonella*. Its expression is enhanced by a truncated IS*Ecp1* element, but the functional consequences of *ramAp* acquisition remain poorly characterized. In this study, we investigated whether *ramAp* modulates chromosomal gene expression in *Salmonella enterica* serovar Typhimurium LT2 and how its expression reshapes bacterial physiology and virulence-associated traits. We first assessed promoter activity using green fluorescent protein (GFP) reporter constructs. The truncated IS*Ecp1* element upstream of *ramAp* significantly increased the expression of downstream genes, supporting its role as a transcriptional enhancer. To identify the direct regulatory targets of RamAp, we focused on genes involved in membrane remodeling (*micF*), oxidative stress response (*sodA*), and motility (*flhDC*), with the efflux pump gene *acrAB* as a known positive control. Electrophoretic mobility shift assays (EMSAs) confirmed that RamAp directly binds to the upstream regulatory regions of all four genes. Functional assays revealed that *ramAp* expression increased SOD enzymatic activity, reduced swimming motility, and enhanced virulence-associated phenotypes in the *Galleria mellonella* infection model. Together, these findings demonstrate that *ramAp* reprograms host gene expression by directly engaging conserved regulatory targets, resulting in the activation of stress adaptation pathways and the repression of motility. Despite its plasmid-borne origin, RamAp functionally overlaps with chromosomal RamA while operating outside the native regulatory constraints, thereby integrating antibiotic resistance, stress resilience, and virulence-associated traits. These results highlight the adaptive potential of horizontally acquired transcriptional regulators under antibiotic pressure and host-associated stress conditions.

## Introduction

The plasmid-encoded transcriptional regulator RamAp was initially identified on IncHI2 multidrug resistance (MDR) plasmids in *Salmonella enterica* and other Gram-negative bacteria. It was first discovered through comparative genomic analysis of two *S. enterica* serovar Goldcoast strains, both of which harbored large plasmids but exhibited distinct antimicrobial resistance phenotypes (Hong et al., 2022). The plasmid from the more resistant strain (R18.0877) contained a 953-bp insertion flanked by insertion sequences, encoding a RamA-like regulator designated *ramAp*. This regulator is identical in nucleotide sequence to chromosomal *ramA* in *Klebsiella*, and the predicted RamAp protein shares 92% amino acid identity with the chromosomal RamA of *Salmonella*, a well-characterized activator of intrinsic multidrug efflux systems (Bailey et al., 2008; Zheng et al., 2009). A multiple sequence alignment further revealed a high degree of conservation between RamAp and chromosomal RamA homologs across representative Enterobacteriaceae species (Supplementary Figure S1). Importantly, *ramAp*-carrying plasmids have since been identified in multiple *Salmonella* and *Klebsiella* genomes available in GenBank, indicating that RamAp is not restricted to the *S. enterica serovar* Goldcoast lineage but is disseminated among members of *Enterobacteriaceae* (Hong et al., 2022; Chiou et al., 2023; Liao et al., 2025; Bernaquez et al., 2025).

In *Salmonella*, the chromosomal *ramA* is negatively regulated by RamR, a transcriptional repressor encoded upstream of *ramA* in a divergent orientation (Abouzeed et al., 2008). In contrast, the plasmid-borne *ramAp* lacks this adjacent regulatory element and is instead preceded by a truncated insertion sequence, ISEcp1*, consisting of a 179-bp fragment corresponding to the right inverted repeat (IRR) of IS*Ecp1*. Similar truncated IS*Ecp1* elements have been identified upstream of resistance genes such as *bla*_CTX-M-15_, *bla*_CMY-42_, and *bla*_OXA-181_, where they enhance downstream gene expression through transcriptional activation (Poirel et al., 2003; Singh et al., 2018; Leelapsawas et al., 2024). Recent studies suggest that IS*Ecp1* not only facilitates the mobilization and expression of resistance genes but may also contribute to their chromosomal integration and long-term stabilization, thereby promoting the persistence and dissemination of multidrug-resistant lineages (Zhang et al., 2025; Shawa et al., 2021). The absence of a dedicated repressor and the presence of the truncated IS*Ecp1* element upstream suggest that *ramAp* expression is likely constitutive or minimally regulated, potentially driven by a cryptic promoter within IS*Ecp1*.

As a homolog of the chromosomal regulator RamA, plasmid-encoded RamAp is expected to interact with components of the same regulatory network in *Salmonella*. RamA belongs to the AraC/XylS family of transcriptional regulators and functions within the *mar*-*sox*-*rob* regulon. In *Klebsiella pneumoniae*, RamA directly activates the *acrAB*-*tolC* multidrug efflux system, contributing to antimicrobial resistance (Nikaido et al., 2008), and RamA-dependent activation of *acrAB* has also been documented in *Salmonella* (Zheng et al., 2011).

Beyond efflux regulation, chromosomal RamA has been implicated in additional cellular processes, including modulation of outer membrane permeability and oxidative stress responses. In *Salmonella enterica* serovar Typhimurium, deletion of *ramA* resulted in reduced expression of *micF*, a type of small regulatory RNA that post-translationally attenuates the major outer membrane porin OmpF (Zheng et al., 2011). Although members of the AcrC/XylS family, such as MarA and Rob, are known to activate *micF* transcription in *Escherichia coli* (Chubiz and Rao, 2011), direct transcriptional regulation of *micF* by RamA has not been conclusively demonstrated, and whether plasmid-borne RamAp influences *micF* expression in *Salmonella* remains unclear.

RamA has also been linked to bacterial oxidative stress tolerance. A *Salmonella* double mutant lacking both *ramA* and *soxRS* exhibits increased sensitivity to superoxide and reduced superoxide dismutase (*sodA*) activity, although no significant defect in early-stage macrophage survival of mouse spleen colonization was observed (van der Straaten et al., 2004). These findings suggest that RamA-mediated oxidative defense may contribute to bacterial fitness under sustained or prolonged stress conditions rather than during acute infection. Whether plasmid-encoded RamAp participates in such stress-adaptive responses has not been examined.

In addition, several MarA/SoxS/Rob family regulators, including RamA, have been shown to negatively influence flagellar gene expression in *Salmonella enterica*, although direct binding of chromosomal RamA to the *flhDC* promoter has not been detected (Thota and Chubiz, 2019). As flagella facilitate motility and host invasion during early infection while simultaneously triggering host innate immune responses, downregulation of flagellar expression is thought to contribute to immune evasion during later stages of infection (Spöring et al., 2018). In this context, it is therefore plausible that plasmid-borne RamAp contributes to motility regulation, potentially through direct interaction with the flhDC promoter under a distinct regulatory framework.

Collectively, these observations indicate that chromosomal RamA influences a range of cellular functions beyond antimicrobial efflux, including envelope remodeling, oxidative stress tolerance, and motility regulation. Our previous study confirmed that the ISEcp1*-*ramAp* region enhances *acrAB* expression and increases antimicrobial resistance in *E. coli* (Hong et al., 2022). However, the broader regulatory and phenotypic consequences of RamAp expression, particularly in the context of stress adaptation and virulence-associated traits, remain incompletely characterized.

To address this, we selected a set of representative target genes associated with key physiological processes previously linked to RamA function. The efflux pump gene *acrAB* was included as a well-established positive control for RamA-mediated activation. In addition, *micF*, a type of small regulatory RNA involved in outer membrane permeability, and *sodA*, encoding superoxide dismutase, were selected to assess potential roles in membrane remodeling and oxidative stress response, respectively. Finally, *flhDC*, the master regulator of flagellar biosynthesis, was examined to evaluate the potential impact of RamAp on motility and virulence-associated traits. Together, these targets provide a focused framework to investigate whether plasmid-borne RamAp can engage conserved regulatory pathways beyond antimicrobial resistance and extend its regulatory influence to broader physiological processes.

In this study, we focused on dissecting the regulatory scope and functional consequences of plasmid-borne RamAp within a defined *Salmonella* genetic background. We evaluated the transcriptional activity of the ISEcp1*-*ramAp* region, examined direct DNA-binding interactions between RamAp and selected chromosomal targets using electrophoretic mobility shift assays (EMSAs), and assessed the impact of *ramAp* expression on bacterial motility, oxidative stress tolerance, and virulence-associated phenotypes using the *Galleria mellonella* infection model as a biologically relevant *in vivo* readout.

## Materials and methods

### Plasmid construction and growth conditions

The *ramAp* gene region used in this study was originally cloned into pMiniT and introduced into *S. enterica* serovar Typhimurium LT2, as previously described (Hong et al., 2022). For stable expression, the *ramAp* region, along with the truncated IS*Ecp1* element upstream, was subcloned into the low-copy-number plasmid pBR322 (p BR322-ISEcp1*-*ramAp*). To assess promoter activity, either a 578-bp truncated IS*Ecp1* element or a 397-bp intergenic region (designated PII) was inserted upstream of GFP in pBR322, generating pBR322-ISEcp1*-GFP and pBR322-PII-GFP. All constructs were verified using Sanger sequencing and introduced into LT2 by electroporation.

Ampicillin (100 μg/mL) was used for selecting plasmids in LT2. Unless otherwise stated, cultures were grown in LB broth at 37 °C.

### GFP reporter assays

GFP fluorescence was measured over a 5-h period in LT2 strains carrying pBR322-GFP, pBR322-ISEcp1*-GFP, or pBR322-PII-GFP. The cultures were grown to mid-log phase (OD₆₀₀ ≈ 0.4) and analyzed using a fluorescence plate reader. Relative fluorescence units (RFUs) were normalized to OD₆₀₀ after background subtraction.

### Expression and purification of RamAp

The *ramAp* gene was cloned into the expression vector pET30 and transformed into *E. coli* BL21 (DE3). Protein expression was induced with IPTG, and RamAp was predominantly expressed as inclusion bodies. The protein was solubilized in 4 M urea and purified via nickel-affinity chromatography, followed by dialysis for refolding.

Kanamycin (50 μg/mL) was used for plasmid selection in *E. coli*.

### Electrophoretic mobility shift assays

Binding reactions (10 μL) contained 10 ng of target DNA (e.g., *micF* and *sodA*), recombinant RamAp (0–600 ng), and binding buffer. After 30 min of incubation at room temperature, the samples were resolved by native PAGE and stained with SYBR™ Safe. DNA-protein complexes were visualized using a gel imaging system to assess the binding activity.

### Superoxide dismutase assay

SOD activity was measured in LT2 strains carrying pBR322-ISEcp1*-*ramAp* or an empty vector control. Crude lysates were prepared and analyzed using the CheKine™ Micro Superoxide dismutase (SOD) Activity Assay Kit (Abbkine, Atlanta, GA, USA), following the manufacturer’s instructions. Activity values were normalized to total protein concentration.

### Motility assays and single-cell tracking

Swimming motility was assessed on TSA/ampicillin plates containing 0.5% agar. For single-cell swimming analysis, bacterial cultures were grown in TB broth and washed with motility buffer (MB) as described (Lin et al., 2021). Cells were loaded into a microchannel formed by double-sided tape between a glass slide and a pre-cleaned coverslip and sealed with petroleum jelly to prevent evaporation.

Videos were recorded under phase-contrast illumination using a Nikon Diaphot 200 microscope equipped with a 10 × eyepiece and 20 × objective, coupled to a Google Pixel 7a smartphone at 3 × zoom. Before analysis, videos were pre-processed using ffmpeg to adjust the frame rate to 30 fps, resize the resolution to 1,228 × 922 pixels, and remove audio.

Pixel-to-micron calibration was performed by imaging a Neubauer counting chamber under identical conditions, yielding a scale of 1 μm ≈ 3.568 pixels. Bacterial trajectories were analyzed using the YSMR software (Schwanbeck et al., 2020). Tracking parameters in the configuration file (tracking.ini) were manually adjusted to optimize bacterial detection based on multiple test runs.

### *Galleria mellonella* infection model

*Galleria mellonella* larvae (250 ± 20 mg) were injected with 10 μL of bacterial suspension at concentrations ranging from 10^5^ to 10^7^ CFU and incubated at 37 °C. Based on preliminary experiments (Supplementary Figure S2), a dose of 1 × 10^6^ CFU per larva was selected as optimal for detecting differences in virulence without causing nonspecific lethality. Larval health scores were assessed 24 h post-infection based on pigmentation, motility, and responsiveness, using the scoring criteria previously described (Loh et al., 2013). Each larva was assigned a health index ranging from 0 (dead) to 10 (fully healthy), with intermediate scores reflecting degrees of melanization, reduced activity, and delayed response to touch.

## Results

### Truncated IS*Ecp1* enhances downstream gene expression in *Salmonella*

**Figure 1:**
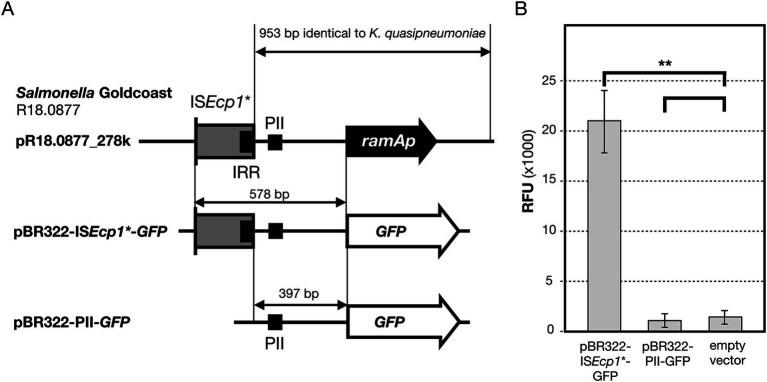
Truncated IS*Ecp1* enhances downstream gene expression in *Salmonella.* **(A)** Schematic representation of the native *ramAp* locus on the large plasmid pR18.0877 of *Salmonella enterica* serovar Goldcoast (top), and two GFP reporter constructs used to assess transcriptional activity of upstream sequences (middle and bottom). The construct pBR322-ISEcp1*-GFP contained a 578-bp fragment encompassing the truncated IS*Ecp1* (ISEcp1*) element and the upstream region of *ramAp*. The construct pBR322-PII-GFP contained a 397-bp non-coding region (termed PII) between ISEcp1* and the *ramAp* start codon. **(B)** GFP fluorescence (relative fluorescence units, RFUs) measured in *S. Typhimurium* LT2 carrying each construct. Only the ISEcp1*-containing construct led to strong GFP expression (~20,000 RFU), while both the PII-only and promoterless controls exhibited minimal fluorescence. Bars represent the mean of biological triplicates; error bars indicate standard deviation. Statistical significance was assessed using unpaired two-tailed *t*-tests. A significant difference was observed between the ISEcp1* and control groups (**p < 0.0001), while the PII and control groups did not differ significantly. ** indicates *p* < 0.0001.

To assess the transcriptional activity of the truncated IS*Ecp1* element upstream of *ramAp*, we constructed a series of green fluorescent protein (GFP) reporter plasmids in *S. enterica* serovar Typhimurium LT2 (Figure 1). The construct pBR322-ISEcp1*-GFP included a 578-bp fragment containing the truncated IS*Ecp1* element (ISEcp1*) and the upstream non-coding region of *ramAp*. A second construct, pBR322-PII-GFP, carried a 397-bp intergenic region corresponding to a putative *ramA* promoter region previously identified in *Klebsiella pneumoniae* based on RACE mapping (Rosenblum et al., 2011). A promoterless plasmid (pBR322-GFP) served as a negative control.

LT2 cells harboring pBR322-ISEcp1*-GFP exhibited strong GFP fluorescence, indicating transcriptional enhancer activity of the truncated IS*Ecp1* element. In contrast, fluorescence levels in strains carrying pBR322-PII-GFP or the promoterless control remained at baseline. These results show that the truncated IS*Ecp1* fragment activates downstream gene expression under the tested conditions.

### RamAp directly binds upstream regulatory regions of target genes

**Figure 2:**
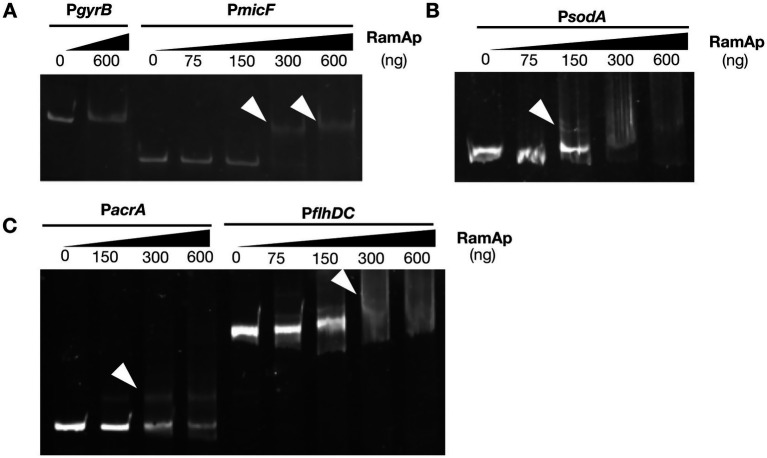
Direct binding of RamAp to the regulatory regions of target genes. Electrophoretic mobility shift assays (EMSAs) were performed to assess binding of recombinant RamAp to upstream regulatory regions of candidate target genes. Increasing amounts of purified RamAp protein (0 ~ 600 ng) were incubated with PCR- amplified DNA fragments and resolved on native agarose gels. The amplified regions and their lengths were as follows: *acrA* (381 bp), *micF* (252 bp), *gyrB* (349 bp), *flhDC* (588 bp), and *sodA* (371 bp). Primer sequences are listed in Supplementary Table S1. **(A)** EMSA results for *gyrB* and *micF*. **(B)** EMSA results for *sodA*. **(C)** EMSA results for *acrA* and *flhDC*. White arrowheads indicate shifted DNA–protein complexes. Full EMSA datasets are provided in Supplementary Figure S3.

The selected target regions and their predicted RamA-binding status, as reported in previous studies (Middlemiss et al., 2023), together with their evaluation in this study, are summarized in Supplementary Table S1. To determine whether RamAp directly interacts with the upstream regulatory regions of stress response and motility genes, we performed EMSAs using purified recombinant RamAp and PCR-amplified DNA fragments (Figure 2). Binding was evidenced by the appearance of shifted bands corresponding to DNA–protein complexes.

As shown in Figure 2A, RamAp bound to the upstream regulatory regions of *acrA* (PacrA) and *micF* (PmicF). As shown in Figure 2B, specific binding to the upstream region of *sodA* (PsodA) was observed. Figure 2C shows binding to the upstream region of *flhDC* (PflhDC), the master regulator of flagellar biosynthesis.

No shift was detected for the *gyrB* upstream region (P*gyrB*), which served as a negative control. P*acrA* was included as a positive control in multiple gels and consistently exhibited strong binding. Full EMSA datasets are provided in Supplementary Figure S3. Together, these results show that RamAp binds to the upstream regulatory regions of *acrA*, *micF*, *sodA,* and *flhDC*.

### RamAp expression increases superoxide dismutase activity

**Figure 3:**
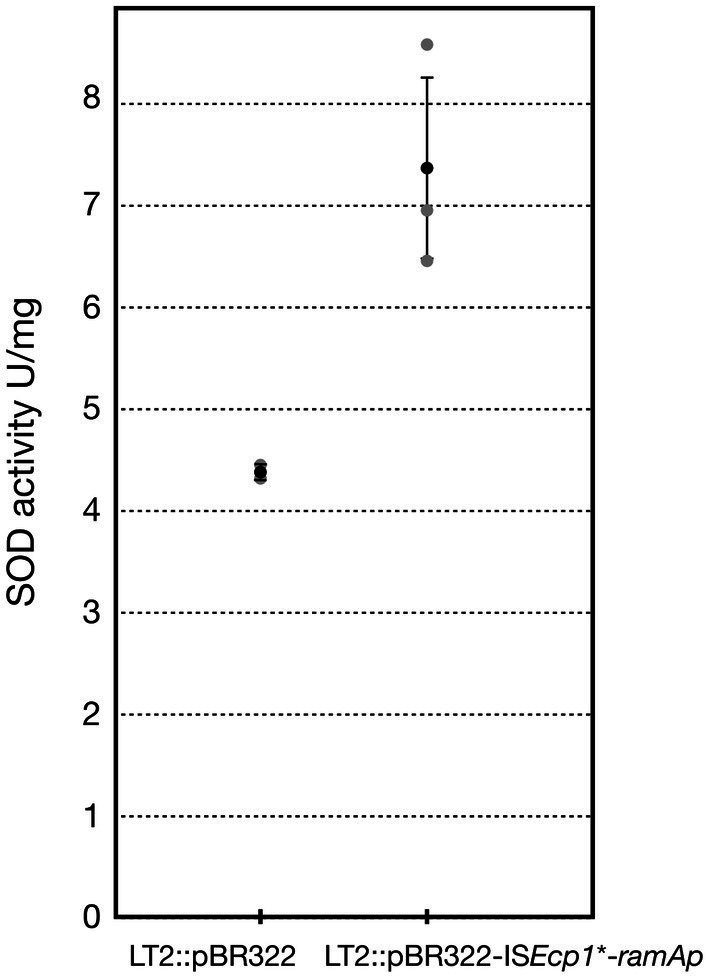
RamAp enhances superoxide dismutase (SOD) activity in *Salmonella* LT2. SOD activity was measured in crude lysates of *S. enterica* serovar Typhimurium LT2 carrying either pBR322 or pBR322-ISEcp1*-*ramAp* using a commercial assay kit. Activity values were normalized to total protein concentration. Each data point represents an independent biological replicate (*n* = 3); horizontal lines indicate the mean and standard deviation. A significant increase in SOD activity was observed in the *ramAp*-expressing strain.

To assess whether RamAp affects superoxide dismutase (SOD) activity, total SOD levels were measured in *S. enterica* serovar Typhimurium LT2 strains carrying either pBR322 or pBR322-ISEcp1*-*ramAp*. Under standard growth conditions on TSA plates supplemented with ampicillin, the *ramAp*-expressing strain exhibited significantly higher SOD activity compared to the control (Figure 3). Each measurement was normalized to total protein content, and the values represent biological triplicates.

### RamAp suppresses swimming motility in *Salmonella* LT2

**Figure 4:**
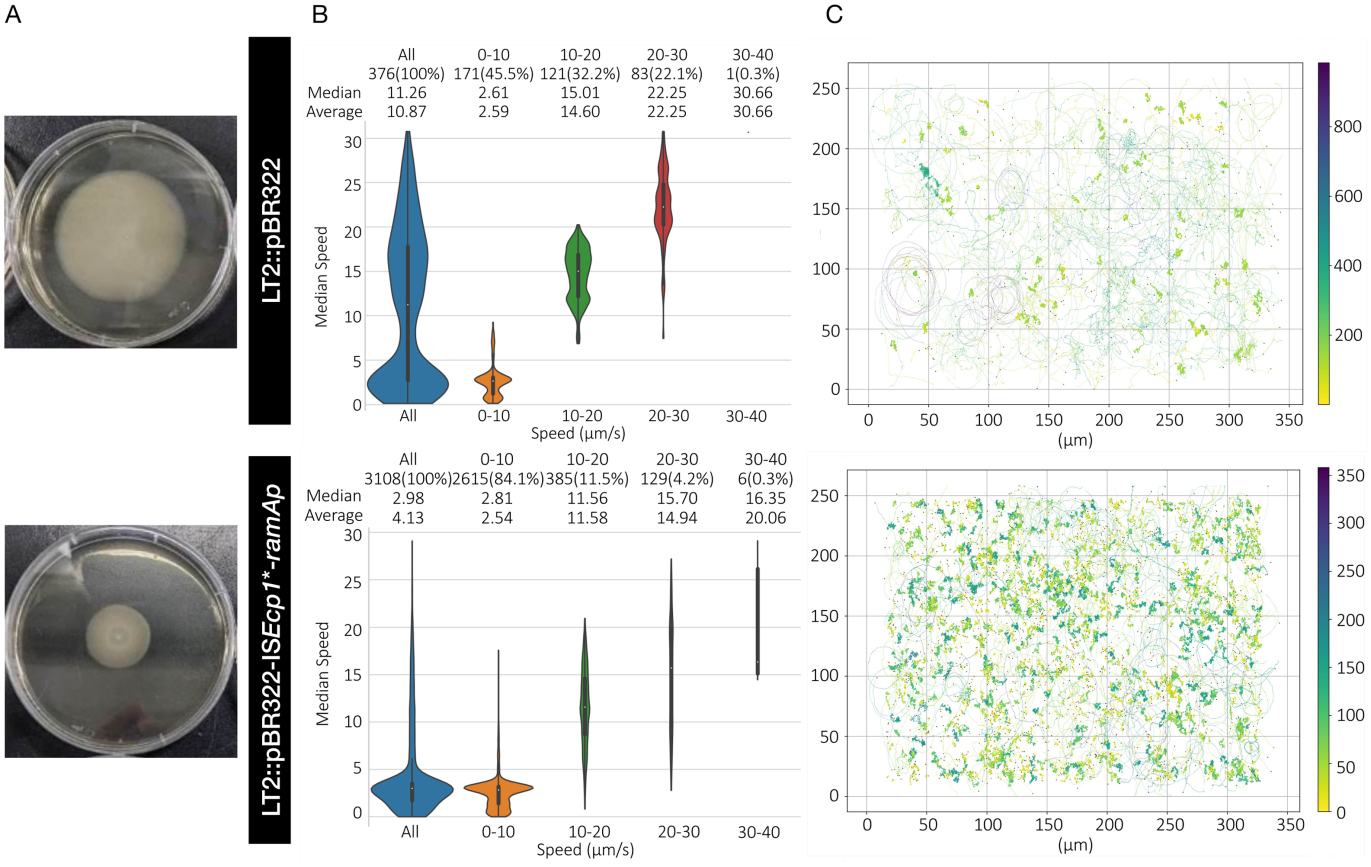
RamAp expression reduces swimming motility in *Salmonella* LT2. Motility was assessed in *S. enterica* serovar Typhimurium LT2 strains carrying either pBR322-ISEcp1*-*ramAp* (top row) or the empty vector pBR322 (bottom row). **(A)** Representative images of swim plates (TSA + ampicillin + 0.3% agar) after incubation at 37 °C for 5 h. **(B)** Median swimming speeds of individual bacterial cells analyzed by phase-contrast time-lapse microscopy and quantified using the YSMR software. **(C)** Overview of single-cell tracks from the same recordings. Colors represent the distance of travel (μm). In the pBR322-ISEcp1*-*ramAp* group, most trajectories appear as stationary green dots due to Brownian motion of non-motile cells.

To assess the impact of RamAp on bacterial motility, we evaluated swimming behavior in *S. enterica* serovar Typhimurium LT2 strains carrying either pBR322-ISEcp1*-*ramAp* or the control plasmid (pBR322). On 0.3% TSA soft agar plates, the RamAp-expressing strain exhibited markedly reduced diffusion zones compared to the control (Figure 4A).

Single-cell motility profiles were further analyzed using the YSMR tracking system (Figure 4B). In the control strain (LT2::pBR322), the average median swimming speed was 11.26 μm/s. Among individual cells, 32.2% had speeds in the range of 10–20 μm/s, 22.1% in 20–30 μm/s, and 0.3% exceeded 30 μm/s. In contrast, the *ramAp*-expressing strain (LT2::pBR322-ISEcp1*-*ramAp*) exhibited a much lower average median speed of 2.98 μm/s, with over 80% of cells swimming at less than 10 μm/s. Only 11.5 and 4.2% of cells in this group fell within the 10–20 μm/s and 20–30 μm/s ranges, respectively. The reduced motility in the *ramAp*-expressing population was also evident in reconstructed tracks, showing a high proportion of cells with minimal displacement (Figure 4C).

### RamAp enhances virulence in a *Galleria mellonella* infection model

To evaluate whether *ramAp* affects the virulence of *Salmonella*, we used the *Galleria mellonella* larval infection model, which provides a simple and reproducible platform for assessing bacterial pathogenicity *in vivo*. Larvae were injected with 1 × 10^6^ CFU of *S. enterica* serovar Typhimurium LT2 carrying either the control plasmid (pBR322) or ramAp-expressing plasmid (pBR322-ISEcp1*-*ramAp*).

**Figure 5:**
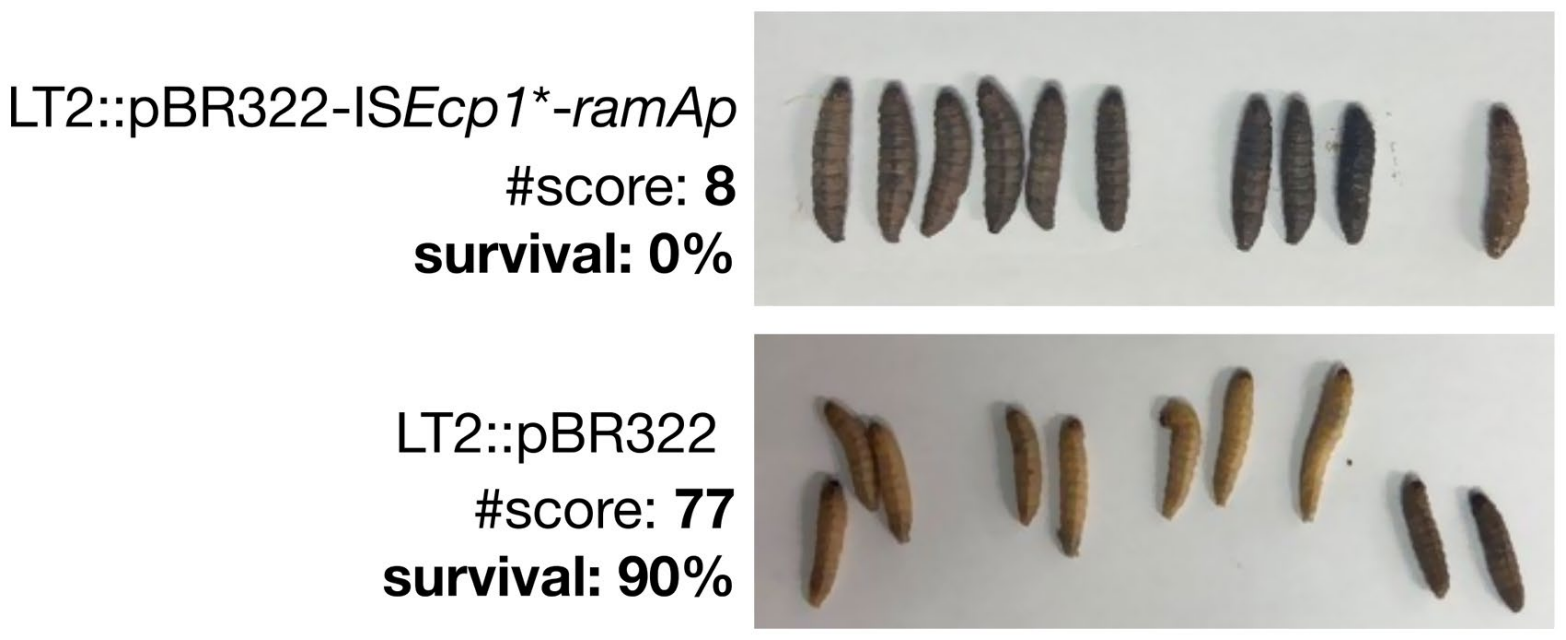
RamAp enhances virulence in *Salmonella enterica*, as shown in the *Galleria mellonella* infection model. Representative images of *Galleria mellonella* larvae 24 h post-infection with *Salmonella enterica* serovar Typhimurium LT2 carrying either the control plasmid pBR322 (bottom) or pBR322-ISEcp1*-*ramAp* (top). Each group consisted of 10 larvae with an average weight of ~192.7 mg. Larvae were injected with 1 × 10^6^ CFU per individual. The pBR322-ISEcp1*-*ramAp* group showed 0% survival and a total health score of 8 (out of 100), indicating complete melanization and loss of motility. In contrast, the control group showed 90% survival and a total health score of 77, with most larvae remaining active and lightly pigmented. Health scores were determined based on pigmentation, movement, and responsiveness, as described by Loh et al. (2013).

At 24 h post-infection, all larvae infected with the *ramAp*-expressing strain showed complete melanization, loss of movement, and 0% survival, resulting in a total health score of 8 out of 100 (Figure 5, top panel). In contrast, 90% of the larvae injected with the control strain survived and retained motility and light pigmentation, with a corresponding health score of 77 (Figure 5, bottom panel).

These results show that the *ramAp*-expressing strain exhibited reduced survival in *Galleria mellonella* larvae compared to the control.

## Discussion

This study demonstrates that the plasmid-encoded gene *ramAp*, a homolog of chromosomal *ramA*, encodes a transcriptional regulator that contributes to both antibiotic resistance and virulence-associated phenotypes in *Salmonella*. Despite its horizontal acquisition, RamAp retains the capacity to engage conserved regulatory circuits in the host bacterium, modulating core physiological functions under a plasmid-borne regulatory context.

Our data show that the truncated IS*Ecp1* element located upstream of *ramAp* functions as a transcriptional enhancer, as evidenced by significantly elevated GFP expression in reporter assays. This enhancer-like activity is consistent with previous observations that truncated IS*Ecp1* elements can drive high-level expression of downstream resistance genes, including β-lactamase genes, in other Enterobacteriaceae (Poirel et al., 2003). In the absence of chromosomal repressors such as RamR and in the presence of the truncated IS*Ecp1* element, *ramAp* expression is likely released from native regulatory constraints. This may result in elevated and potentially dysregulated expression with broad downstream consequences.

Although a promoter region upstream of chromosomal *ramA* has been proposed in *Klebsiella pneumoniae*, notably the PII fragment identified by EMSA as a RamR- and RamA-binding site (Rosenblum et al., 2011), our results indicate that this region alone does not exhibit detectable promoter activity in *Salmonella*. We cloned a 397-bp fragment from the plasmid-borne *ramAp* locus in LT2, which corresponds to the PII region in *Klebsiella*, into a GFP reporter construct, but the observed GFP signal was indistinguishable from the promoterless control, suggesting minimal or no detectable promoter activity (Figure 1). This suggests that, despite its capacity to bind transcriptional regulators, the PII-like sequence may not function as an autonomous promoter in this genetic background. Alternatively, its activity may depend on additional cis-regulatory elements, chromosomal context, or high-order DNA architecture, all of which are absent in the plasmid-derived construct.

EMSAs confirmed that RamAp binds directly to the upstream regulatory regions of *acrAB* and *micF*, consistent with its function as a transcriptional regulator (Figure 2A). These targets overlap substantially with previously characterized components of the RamA regulon, supporting the notion that RamAp can access conserved regulatory sites in *Salmonella enterica*. RamA was first shown to activate *acrB* expression by Bailey et al. (2008) and was later found to upregulate *acrAB*, *acrEF*, and *tolC* upon overexpression in *S. enterica* serovar Typhimurium SL1344 (Bailey et al., 2010). Middlemiss et al. (2023) further confirmed *acrA* and *micF* as RamA-bound targets using ChIP-seq analysis. Our EMSA results are consistent with these studies and extend them by demonstrating direct DNA binding by a plasmid-encoded RamA homolog.

The regulatory influence of RamAp extends beyond efflux and membrane remodeling to include oxidative stress response. We observed increased *sodA* expression and elevated superoxide dismutase (SOD) activity in RamAp-expressing strains (Figure 3), indicating enhanced capacity to detoxify reactive oxygen species. Although *sodA* was not identified as a RamA-bound target in recent ChIP-seq analyses (Middlemiss et al., 2023), an earlier study by van der Straaten et al. (2004) demonstrated that chromosomal *ramA* contributes to oxidative stress tolerance in *Salmonella,* in part through *sodA* activation. Our findings suggest that RamAp can recapitulate this stress-associated regulatory function, further integrating into host adaptation responses.

The regulatory effects of RamAp also extend to motility-related pathways. In both soft agar swimming assays and single-cell tracking analyses, we observed significantly reduced swimming behavior in RamAp-expressing strains (Figure 4). This phenotype correlates with reduced *flhDC* expression and with direct RamAp binding to the *flhDC* upstream region, as shown by EMSA (Figure 2C). Together, these results support a direct role for RamAp in transcriptional repression of motility.

In *Salmonella*, *flhDC* encodes the class I master regulators at the apex of the flagellar gene hierarchy, controlling downstream class II and III genes required for flagellar assembly and function (Chilcott and Hughes, 2000). Repression of *flhDC* by chromosomal RamA has been reported previously: Thota and Chubiz (2019) showed that RamA overexpression results in reduced *flhDC* expression and impaired motility, although direct DNA binding was not detected in that context. Our data demonstrate that a plasmid-borne RamA homolog, expressed under a distinct regulatory regime, is capable of directly interacting with the *flhDC* promoter, providing a mechanistic basis for motility expression not previously observed for chromosomal RamA.

One plausible explanation for this regulatory outcome involves a physiological trade-off in proton motive force (PMF) allocation. In *Salmonella enterica* serovar Typhimurium, Lyu et al. (2021) demonstrated that flagellated cells (fliC-ON) exhibit higher intracellular proton concentrations and reduced antibiotic tolerance, likely due to competition between flagellar rotation and TolC-dependent efflux systems for PMF. Similar trade-offs have been reported in *Pseudomonas aeruginosa*, where activation of RND efflux systems suppresses flagellar gene expression through alterations in intracellular pH (Lembke et al., 2025). These observations support a model in which RamAp-mediated repression of motility reallocates energetic resources toward efflux activity and stress resistance, enhancing bacterial survival under antibiotic pressure.

More broadly, our findings support the hypothesis that RamAp expression induces coordinated physiological reprogramming in *Salmonella*, characterized by suppression of energetically costly dispersal functions and reinforcement of survival-associated traits. This interpretation is consistent with prior observations that deletion of *acrD* leads to increased *flhDC* expression and enhanced motility (Buckner et al., 2016), underscoring an intrinsic regulatory tension between motility and resistance. Importantly, our results demonstrate that this balance can be shifted by a horizontally acquired transcriptional regulator. In line with this, mobile genetic elements have been increasingly recognized as important contributors to the coordination of virulence and resistance traits through regulatory and horizontal gene transfer mechanisms (Li and Farzana, 2026). This highlights the capacity of plasmid-encoded regulators to rewire host regulatory hierarchies.

Taken together, our findings indicate that RamAp, although plasmid-encoded and likely derived from *Klebsiella*, retains the ability to engage conserved transcriptional targets in *Salmonella*, including *acrAB*, *micF*, *sodA*, and *flhDC*. By directly binding to these regulatory regions, RamAp functionally overlaps with chromosomal RamA while operating outside native regulatory constraints, effectively acting as an unrestrained analog with broad transcriptional influence.

Notably, in our previous study, the introduction of *ramAp* into *E. coli* led to increased efflux pump expression and MDR, and *ramAp*-carrying plasmids have been identified across multiple Enterobacteriaceae species. These findings support the potential for similar regulatory effects across diverse bacterial hosts (Hong et al., 2022).

Consistent with this regulatory profile, RamAp expression enhanced virulence-associated phenotypes in the *Galleria mellonella* infection model. Larvae infected with RamAp-expressing strains exhibited increased mortality and reduced health scores, reflecting enhanced bacterial fitness under host-mimicking stress conditions rather than direct immune modulation. This phenotype may, in part, reflect an enhanced ability to withstand host immune defenses. These findings support the interpretation that RamAp promotes traits associated with persistence and stress tolerance during infection.

While this study provides important insights into the regulatory role of RamAp, several limitations should be acknowledged. First, our analysis focused on a selected set of candidate genes rather than a global transcriptomic approach. Although we demonstrated direct binding of RamAp to key regulatory regions and validated their functional consequences through biochemical and phenotypic assays, the absence of transcriptome-wide data limits our ability to comprehensively define the full regulatory network governed by RamAp. Future studies employing RNA sequencing or ChIP-seq analyses would provide a more systematic view of RamAp-dependent gene regulation and may reveal additional downstream targets.

Second, all functional analyses were conducted in a defined *S. enterica* serovar Typhimurium LT2 background. While this controlled system allowed us to dissect the mechanistic roles of RamAp, it may not fully capture the diversity of regulatory outcomes in other genetic backgrounds or host species. Given the interspecific conservation of RamAp and its plasmid-borne nature, further investigation in clinically relevant strains and additional bacterial species will be important to evaluate the broader impact of this regulator.

Finally, although the *Galleria mellonella* infection model provides a convenient and reproducible platform for assessing virulence-associated phenotypes, it does not fully recapitulate the complexity of mammalian host–pathogen interactions. Additional *in vivo* studies using mammalian infection models would be valuable to further validate the contribution of RamAp to pathogenicity.

Unlike conventional resistance determinants that encode discrete enzymatic defenses, RamAp functions by reprogramming host regulatory networks, thereby integrating resistance and virulence traits in a single horizontally acquired regulator. Its recurrent association with MDR plasmids underscores its adaptive value, particularly in environments where antibiotic pressure and host-associated stresses converge. In this context, RamAp represents a distinct class of plasmid-borne transcriptional regulators capable of reshaping bacterial physiology from within.

## Data Availability

The datasets presented in this study can be found in online repositories. The names of the repository/repositories and accession number(s) can be found in the article/Supplementary material.
