# Supplementary material for: Plasmid-borne transcriptional regulator RamAp modulates Salmonella genes for environmental and host adaptation
Source: Front Microbiol. 2026 May 28;17:1842592. doi: 10.3389/fmicb.2026.1842592 (PMC13253676; doi:10.3389/fmicb.2026.1842592)
Supplement: Supplementary file 1 [file Data_Sheet_1.pdf]

Supplementary materials

Fig. S1. Multiple sequence alignment of RamAp and representative chromosomal RamA homologs

Multiple sequence alignment of RamAp and representative chromosomal RamA homologs from Enterobacteriaceae. Amino acid sequences were aligned using ClustalW. The alignment includes RamAp from this study and chromosomal RamA proteins from *Salmonella enterica* serovar Goldcoast (strain R18.0877), *Klebsiella quasipneumoniae* (ATCC 700603), *Klebsiella pneumoniae* (MGH78578), *Enterobacter aerogenes* sp. 638, and *Citrobacter koseri* (ATCC BAA-895). Conserved residues are indicated.

```
ramAp                       MTISAQVIDTIVEWIDDNLHQPLRIDDIARHAGYSKWHLQRLFLQYKGES
ramA_K.quasipneumoniae      MTISAQVIDTIVEWIDDNLHQPLRIDDIARHAGYSKWHLQRLFLQYKGES
ramA_K.pneumoniae           MTISAQVIDTIVEWIDDNLHQPLRIDDIARHAGYSKWHLQRLFLQYKGES
ramA_Enterobacter           MNISAQVIDTIVEWIDDNLHQPLRIDDIARHAGYSKWHLQRLFLQYKGES
ramA_Citrobacter            MTISAQVIDTIVEWIDDNLNQPLRIDDIARHAGYSKWHLQRLFMQYKGES
ramA_Salmonella             MTISAQVIDTIVEWIDDNLNQPLRIDDIARHAGYSKWHLQRLFMQYKGES
                            *.*****************:***********************:******

ramAp                       LGRYIRERKLLLAARDLRDTDQRVYDICLKYGFDSQQTFTRVFTRTFNLP
ramA_K.quasipneumoniae      LGRYIRERKLLLAARDLRDTDQRVYDICLKYGFDSQQTFTRVFTRTFNLP
ramA_K.pneumoniae           LGRYIRERKLLLAARDLRDTDQRVYDICLKYGFDSQQTFTRVFTRTFNQP
ramA_Enterobacter           LGRYIRERKLLLAARDLRDTDQRVYDICLKYGFDSQQTFTRIFTRTFNQP
ramA_Citrobacter            LGRYIRERKLRLAARDLRDTDQRVYDICLKYGFDSQQTFTRIFTRTFNQP
ramA_Salmonella             LGRYVRERKLKLAARDLRDTDQKVYDICLKYGFDSQQTFTRIFTRTFNLP
                            ****:***** ************:*****************:****** *

ramAp                       PGAYRKENHSRAH
ramA_K.quasipneumoniae      PGAYRKENHSRAH
ramA_K.pneumoniae           PGAYRKENHSRAH
ramA_Enterobacter           PGAYRKENHSRAH
ramA_Citrobacter            PGAYRKENHSRTH
ramA_Salmonella             PGAYRKEKHGRTH
                            *******:*.*:*
```

Fig. S2. Identification of an optimal infection dose for assessing RamAp-mediated virulence in *Galleria mellonella*.

To determine a suitable inoculum for differentiating the virulence of *Salmonella enterica* serovar Typhimurium LT2 strains carrying either pBR322 or pBR322-IS*Ecp1**-*ramAp*, *G. mellonella* larvae were injected with 1×10^5, 1×10^6, or 1×10^7 CFU per larva. Each panel shows a dish containing 10 larvae imaged 24 hours post-infection. At 1×10^5 CFU, all larvae remained healthy in both groups, whereas at 1×10^7 CFU, high mortality occurred regardless of plasmid background. In contrast, infection with 1×10^6 CFU revealed a distinct difference in larval survival and melanization between the two strains, making it the most appropriate dose for comparative virulence assays (as shown in Fig. 5). Images shown here are from an independent replicate of the experiment presented in the main figure.

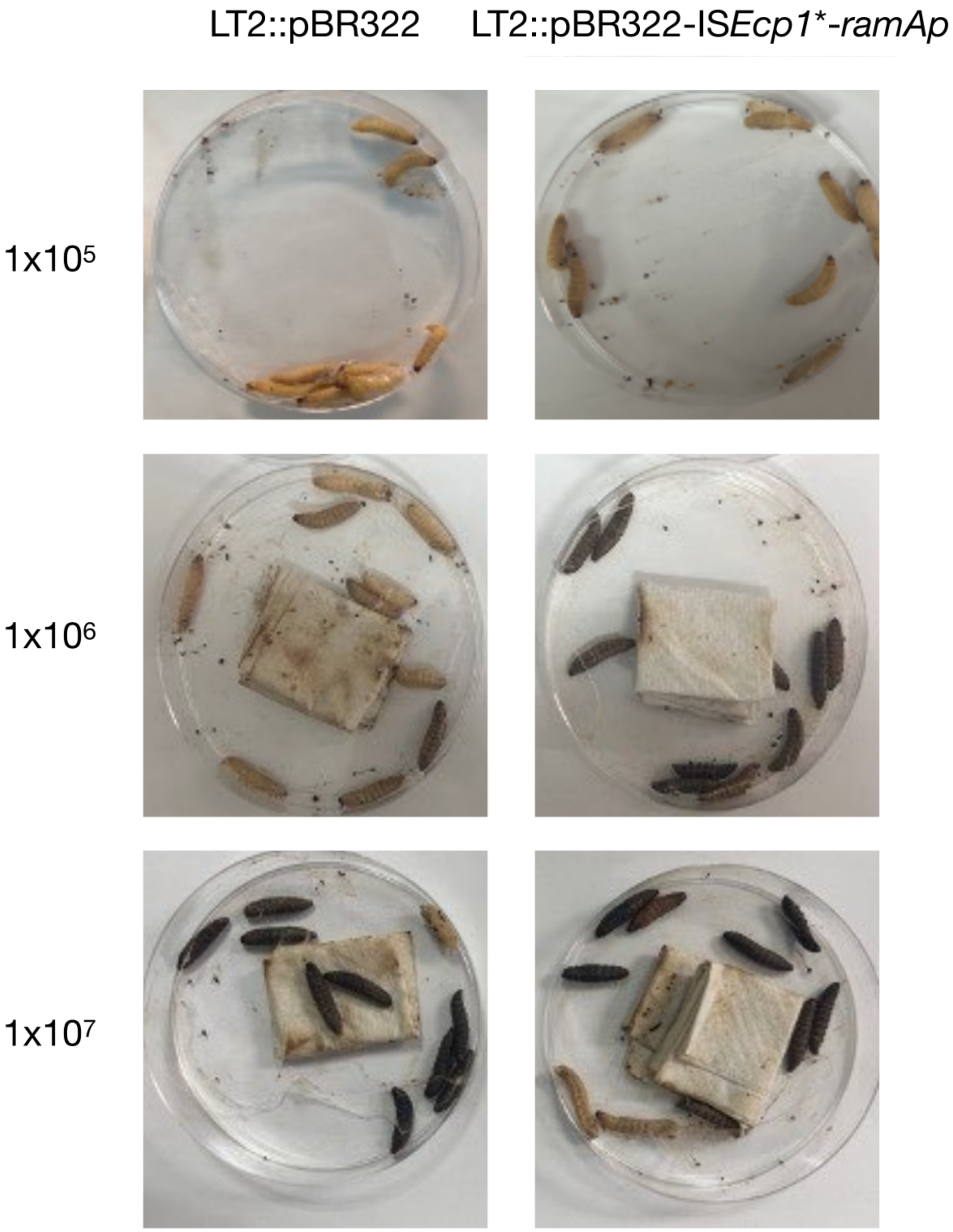
LT2::pBR322
LT2::pBR322-IS*Ecp1*-ramAp*
1x10^5
1x10^6
1x10^7

Fig. S3. Full EMSA datasets corresponding to Fig. 2.

Electrophoretic mobility shift assays (EMSA) showing the complete gel panels used for analysis of RamAp binding to target promoters. Repeated *acrA* panels were included in each independent gel as a positive control during the original experiments. Increasing amounts of purified RamAp protein (0–600 ng) were incubated with PCR-amplified DNA fragments. White arrowheads indicate shifted DNA–protein complexes.

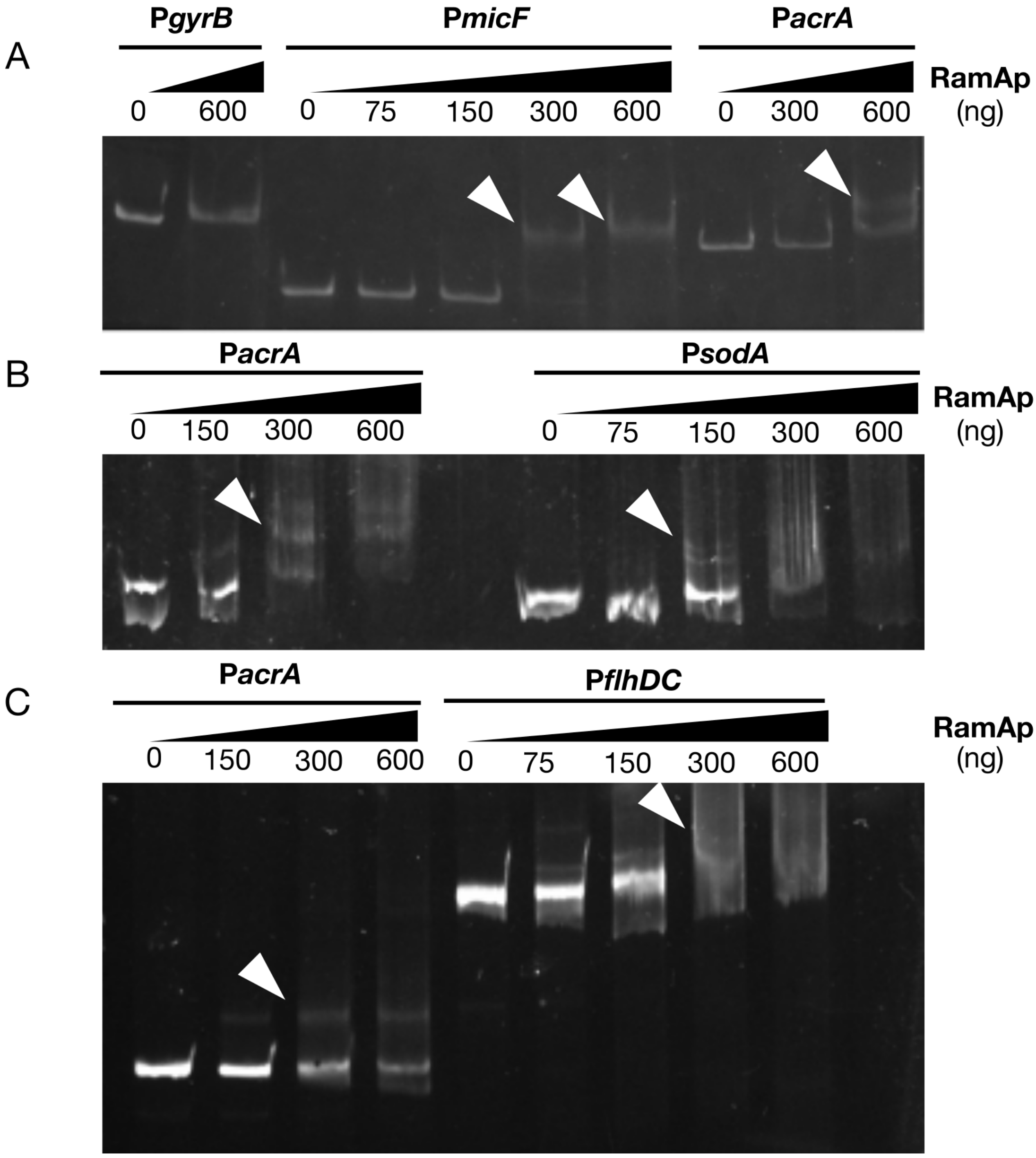

Table S1. Selected target regions for EMSA analysis, their previously reported RamA-binding status, and associated primer information

Target	Amplicon Size	Previously reported RamA-binding status	Primers	Primer Sequences	Tm (°C)
acrA	**381** bp	Yes**	P*acrA*_forward	TGAGAGCATCAGAACGAC	51
			P*acrA*_reverse	CCCAGATCTCACTGAATAAA	
micF	**252** bp	Yes**	P*micF*_forward	CCATTCTCCGCAAAAATACAG	52
			P*micF*_reverse	GCGGTCACTATTTTAGTTGC	
sodA	**371** bp	Not reported	P*sodA*_forward	CCTTCTTGATTTTGCCAC	55
			P*sodA*_reverse	GTTTGATGGTGTTTGGTG	
flhDC	**588** bp	Not reported	P*flhDC*_forward	GTTTGCCATCTCTTCGTT	57
			P*flhDC*_reverse	GTTATCTATTATCCTGGCGT	
gyrB	**349** bp	Negative control	P*gyrB*_forward	CTACCACCTCGAATACCA	51
			P*gyrB*_reverse	CGCTGCCTGTACCTGATA	

Note. PCR conditions were as follows: initial denaturation at 98°C for 30 s; 35 cycles of denaturation at 98°C for 10 s, annealing at primer-specific Tm for 30 s, and extension at 72°C for 30 s; followed by a final extension at 72°C for 10 min. For EMSA assays, *gyrB* was included as a negative control. **Predicted as reported by Middlemiss AD et al. 2023.
